# Supplementary material for: Pseudotyped virus infection of multiplexed ACE2 libraries reveals SARS-CoV-2 variant shifts in receptor usage
Source: bioRxiv. 2024 Feb 14:2024.02.13.580056. Preprint. [Version 2] doi: 10.1101/2024.02.13.580056 (PMC10888787; doi:10.1101/2024.02.13.580056)
Supplement: Supplement 2 [file NIHPP2024.02.13.580056v2-supplement-2.pdf]

bioRxiv preprint doi: https://doi.org/10.1101/2024.02.13.580056; this version posted February 14, 2024. The copyright holder for this preprint (which was not certified by peer review) is the author/funder, who has granted bioRxiv a license to display the preprint in perpetuity. It is made available under aCC-BY 4.0 International license.

Supplementary Figure S1. Comparing SARS-CoV-2 D614G pseudotyped virus infectivity with the multiplex assay as compared to more traditional assay formats. A) Scatter plot showing SARS-CoV-2 D614G spike pseudotyped virus infectivities in cells expressing WT human ACE2, or various human ACE2 mutants, tested through a traditional arrayed infection assay (x-axis) or the multiplex infection assay (y-axis) performed in this work. Infection values were normalized to those from cells expressing WT human ACE2.
B) A) Scatter plot showing SARS-CoV-2 D614G spike pseudotyped virus infectivities in cells expressing human ACE2, or various animal ACE2 orthologs, tested through a traditional arrayed infection assay (x-axis) or the multiplex infection assay (y-axis) performed in this work. Infection values were normalized to those from cells expressing human ACE2.

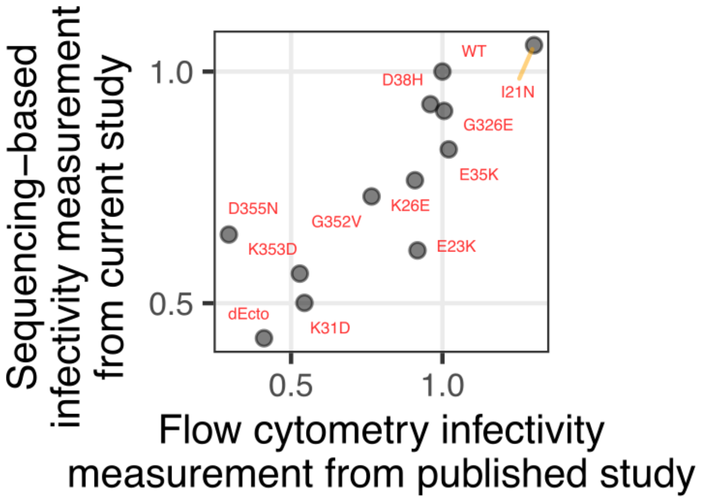

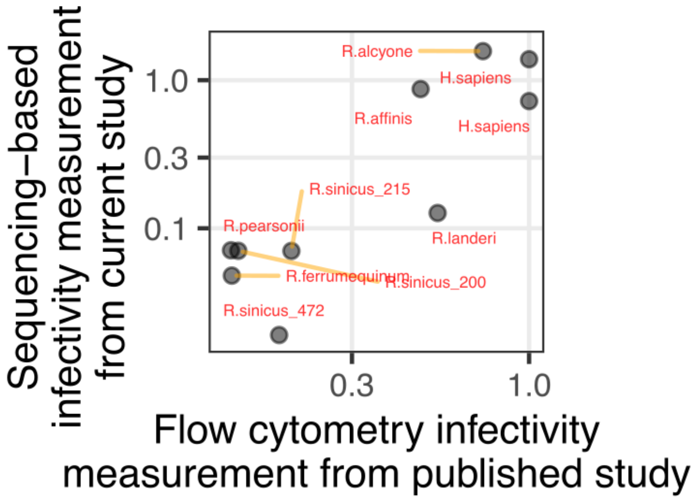

bioRxiv preprint doi: https://doi.org/10.1101/2024.02.13.580056; this version posted February 14, 2024. The copyright holder for this preprint (which was not certified by peer review) is the author/funder, who has granted bioRxiv a license to display the preprint in perpetuity. It is made available under aCC-BY 4.0 International license.

Supplementary Figure S2. Example plots demonstrating the atomic distances between key ACE2 residues and various residues on SARS-CoV-2 spike RBD D614G. A) Scatter plots showing the minimal atomic distance (y-axis) between various SARS-CoV-2 spike RBD D614G residues (x-axis) and human ACE2 residue Lys31 (left), Asp38 (middle), or and overlay of the two for comparison (right). B) Line graphs comparing how the minimal distances for ACE2 Lys31 (left) and Asp38 (right) change in regards to each position on the SARS-CoV-2 spike RBD when ensemble structures from various SARS-CoV-2 variants are used. C) Histogram showing the most variable ACE2:RBD minimum distance pairs, as determined by plotting the pairs with the highest coefficients of variation across SARS-CoV-2 RBD variants. The pairs with the highest values are labeled.

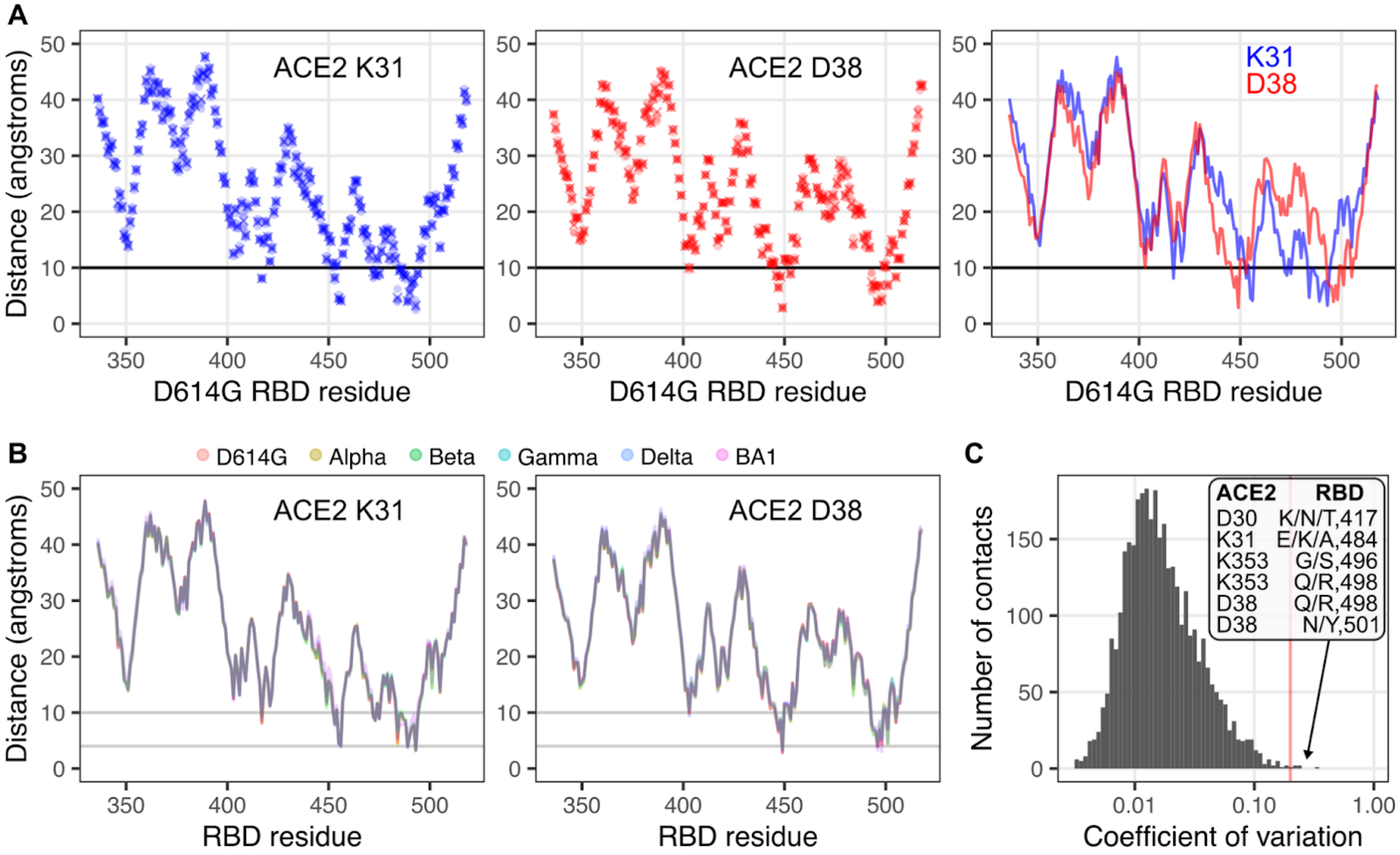

bioRxiv preprint doi: https://doi.org/10.1101/2024.02.13.580056; this version posted February 14, 2024. The copyright holder for this preprint (which was not certified by peer review) is the author/funder, who has granted bioRxiv a license to display the preprint in perpetuity. It is made available under aCC-BY 4.0 International license.

Supplementary Figure S3. Selection scheme for identifying key ACE2 and spike RBD residue pairs with structural analysis. A) Scatterplots comparing atomic distances for each ACE2:RBD residue pair (left), or histograms showing the distribution of difference values that result when the atomic distance of each ACE2:RBD residue pair for one structure was subtracted by the atomic distance of that same pair in a comparative structure (right). Three example pairs of plots comparing atomic distances between D614G spike RBD residues and ACE2 residues, with the same metrics calculated for Beta, Delta, and Omicron BA1 spikes (top). As all 15 pairwise combinations of SARS-CoV-2 variant spike RBD ensemble structures were considered, we have also shown an example pair of plots demonstrating a comparison between Beta and Omicron BA1 spike RBDs (bottom). B) Histogram showing the combined Z-scores of changes in pairwise residue atomic distances for all combinations of SARS-CoV-2 spike RBD ensemble structures. Histogram bars in red denote residue pairs that exhibited values outside of the central 95% interval of the distribution. C) Venn diagram demonstrating how the 38 key residue pairs were chosen for subsequent structural and functional analysis.

bioRxiv preprint doi: https://doi.org/10.1101/2024.02.13.580056; this version posted February 14, 2024. The copyright holder for this preprint (which was not certified by peer review) is the author/funder, who has granted bioRxiv a license to display the preprint in perpetuity. It is made available under aCC-BY 4.0 International license.

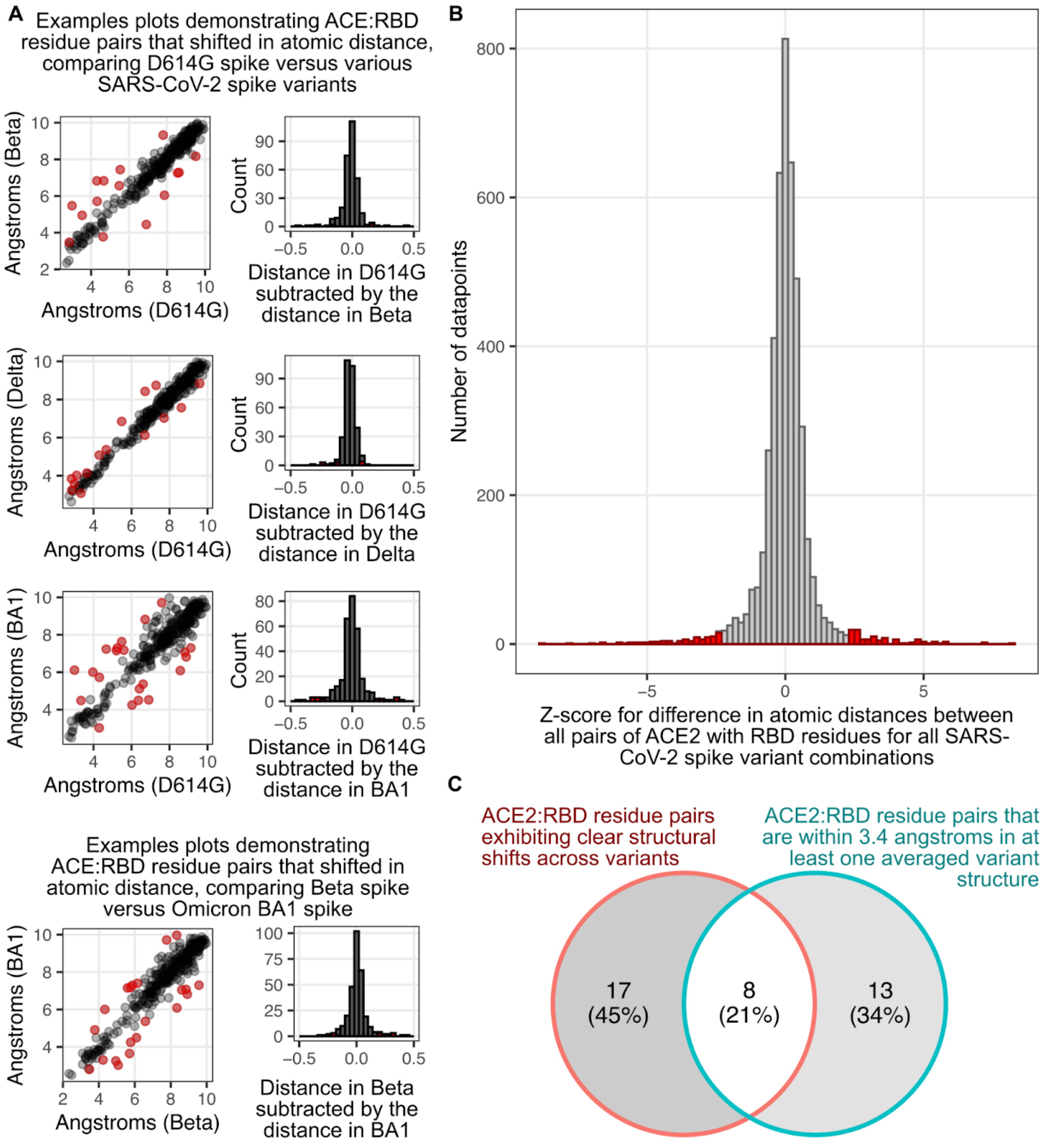

bioRxiv preprint doi: https://doi.org/10.1101/2024.02.13.580056; this version posted February 14, 2024. The copyright holder for this preprint (which was not certified by peer review) is the author/funder, who has granted bioRxiv a license to display the preprint in perpetuity. It is made available under aCC-BY 4.0 International license.

Supplementary Figure S4. Principal component analysis of all ACE2-RBD pairwise distances for all structures analyzed in this work. Scatter plot with the x-axis displaying the first principal component of the ACE2-RBD residue pairwise distance matrix, and the y-axis showing the second principal component. Shapes of points denote method of acquisition, while the color of the point denotes which RBD variant was solved. PDB identifiers are indicated next to each point.

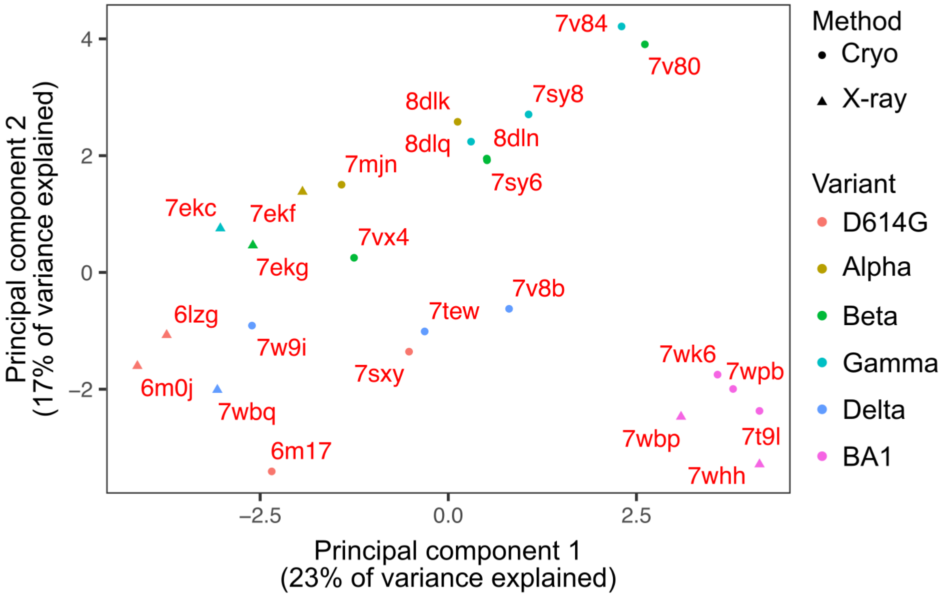

bioRxiv preprint doi: https://doi.org/10.1101/2024.02.13.580056; this version posted February 14, 2024. The copyright holder for this preprint (which was not certified by peer review) is the author/funder, who has granted bioRxiv a license to display the preprint in perpetuity. It is made available under aCC-BY 4.0 International license.

Supplementary Figure S5. Amino acid comparisons between various *R. sinicus* ACE2 alleles. A) Protein sequence comparisons showing residue differences between various chinese horseshoe bat ACE2 alleles, with major differences from the most common amino acid highlighted in red. The human amino acid at each position is shown for comparison, with major differences highlighted in blue. Residues located in the signal peptide or in the first alpha helix of ACE2 are labeled above the curated alignment. B) Tertiary structure of the ACE2 surface interacting with the SARS-CoV-2 spike, highlighting residues that are variable within the R.sinicus alleles. PDB entry 6m17, consisting of a cryo-EM structure of human ACE2 bound with the SARS-CoV-2 spike RBD, was used to show the three-dimensional locations of these highly variable residues. The full ACE2 polypeptide is shown as a cartoon representation, with the amino acid side chains for the human ACE2 protein are shown as spheres. C) Same tertiary structure representation as in panel B, but with the SARS-CoV-2 spike RBD shown as a cyan cartoon representation, including key interaction residues highlighted in our structural analysis shown as spheres.

A

Signal peptide: positions 3–7; Alpha helix 1: positions 24–41

		3	5	7	19	24	27	31	34,35	38	41	84	139	159	316
Rinolophus sinicus alleles tested	ACT66**275**	S	S	L	F	L	I	E	SE	N	Y	P	H	S	V
	ADN93**472**	G	F	F	S	E	I	K	TK	D	H	P	H	N	V
	QMQ39**215**	G	S	L	S	R	T	E	SE	N	Y	S	Q	N	M
	QMQ39**200**	G	S	L	F	L	I	E	FE	N	Y	P	H	N	V
	Human	S	S	L	S	Q	T	K	HE	D	Y	P	Q	N	V

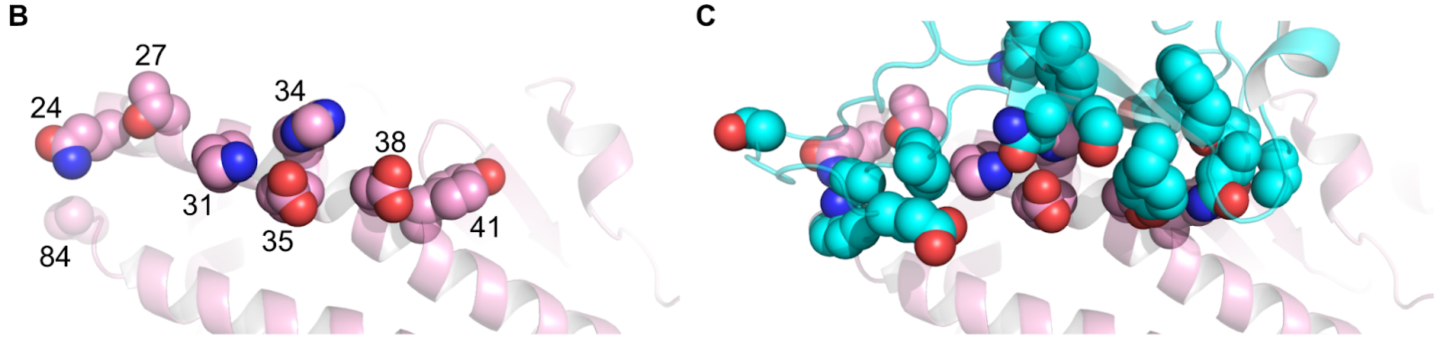

bioRxiv preprint doi: https://doi.org/10.1101/2024.02.13.580056; this version posted February 14, 2024. The copyright holder for this preprint (which was not certified by peer review) is the author/funder, who has granted bioRxiv a license to display the preprint in perpetuity. It is made available under aCC-BY 4.0 International license.

Supplementary Figure S6. Unselected recombined barcode correlations following prolonged passaging or independent freeze-thaws. Set 1 consists of a group of 17 different subpopulations collected as “unselected cells” over the course of two weeks of experiments, derived from the same vial of frozen, recombined cells that were thawed at the start of that series of experiments. Set 2 includes samples derived from an independent thaw of the same library of cells several months later. The 16 “unselected cell” samples were collected over the course of two weeks, spanning December 22, 2022 through January 4, 2023. The histograms show the Pearson’s r-squared values resulting from all pairwise comparisons of all unselected barcode counts sequenced from set 1 or set 2 genomic DNAs, but displayed as different histograms based on whether the correlations were calculated for two samples within the same set or within separate sets.

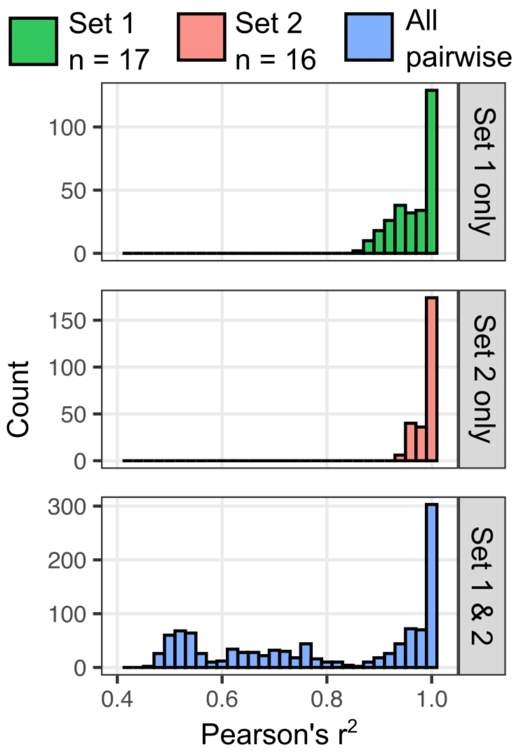

bioRxiv preprint doi: https://doi.org/10.1101/2024.02.13.580056; this version posted February 14, 2024. The copyright holder for this preprint (which was not certified by peer review) is the author/funder, who has granted bioRxiv a license to display the preprint in perpetuity. It is made available under aCC-BY 4.0 International license.

Supplementary table 1: Information for each construct and the primers used to create the barcode

Supplementary table 2: PDB entry identifiers and acquisition types used in the structural analysis

Supplementary table 3: Accession numbers and protein sequences used in this study

Supplementary table 4: Sequencing file names and the sample replicate they corresponded to

Supplementary table 5: Primer names and the partial barcode sequences they encode
